# Clearing the Smokescreen: The Current Evidence on Cannabis Use

**DOI:** 10.3389/fpsyt.2015.00040

**Published:** 2015-03-13

**Authors:** Elizabeth Clare Temple

**Affiliations:** ^1^School of Health Sciences and Psychology, Federation University Australia, Ballarat, VIC, Australia

**Keywords:** cannabis, drug policy, normalization, addiction, psychosis, affective disorders, anxiety

Decisions regarding the legal status of cannabis have long been framed (for the public at least) with reference to the perceived health risks and harms associated with use. Yet, drug policy and legislation relating to the use of cannabis are rarely based on the scientific evidence of the known risks and harms. There are many reasons for this discrepancy, with the politicization of cannabis use, where ideology and moralizing are given precedence over the science, being one. Thus, we begin this research topic with Aggarwal (1) discussion of how such politicization has contributed to the current smokescreen that is obscuring our understanding of cannabis, including the impact it has on the ability of researchers to collect and disseminate accurate information about the effects of cannabis use.

The capacity of policy makers and legislators to develop evidence-based cannabis policies and laws is also contingent on researchers explaining the existing evidence, disseminating new research findings, and collaborating with relevant people, agencies, and government departments to improve the premises on which they base their policies and legislation. Roffman (2), who took this path through his involvement in the development of the legislation to legalize cannabis use in Washington State, provides an insider’s view of the processes and deliberations. While we will have to wait for the evaluation of this carefully designed model for regulating cannabis use, the following two articles provide some insight into patterns of cannabis use in contexts were consumption is relatively normalized. There are many parallels evident in the findings of Mostaghim and Hathaway (3) qualitative exploration of cannabis use among Canadian university students and Liebregts et al. (4) prospective investigation of cannabis use by young adults transitioning from university to work in The Netherlands. Of particular note are the ways in which the participants’ self-identity, including priorities, roles, and responsibilities, act as constraints to their use, and the clear demarcations drawn between leisure and work.

A major consideration, discussed by Roffman (2), was the risk that legalization of cannabis might spark an increase in usage, which could, in turn, result in higher incidence and prevalence of cannabis-related harms, particularly if there was an increase in use by adolescents. The evidence underpinning concerns of adverse impacts resulting from early onset cannabis use is reviewed by Chadwick et al. (5), who report that adolescent users with genetic vulnerabilities are at increased risk of experiencing motivational, affective, and psychotic disorders, including schizophrenia. The association between cannabis and psychosis/schizophrenia is comprehensively reviewed by Radhakrishnan et al. (6), who conclude that, while, cannabis may be a component cause in the development of psychosis, this association is moderated by family history of psychoses, genetic factors, childhood trauma/abuse, and age at onset of use. The importance of differentiating between psychotic disorders and psychomimetic effects is also highlighted as being an essential step in increasing our understanding of the cannabis-psychosis association. Similarly, the two pathways from cannabis use to psychosis proposed by Burns (7) illustrate the importance of differentiating between types of cannabis-psychosis trajectories, showing how the clinical presentation profiles and treatment outcomes differ for early onset, long-term cannabis use in comparison to later onset, short-term but intense use.

Early onset/adolescent cannabis use is investigated further in the next three articles. First, Serafini et al. (8) explore the possible role of hopelessness as a mediator in the relationship between early cannabis use and suicidal behaviors, while Little et al. (9) investigate predictors of cannabis cessation within a sample of high-school students. The next article, by Fallu et al. (10), reports the findings of a latent class analysis of adolescent cannabis users, revealing four different use trajectories. The early onset, heavy cannabis and polydrug use group in this study were found to experience the highest level of use-related problems, followed by the late-heavy-polydrug group. Similarly, Connor et al. (11) report that, in a sample of adult cannabis users referred for treatment, those who engaged in polydrug use were more likely to be cannabis dependent and experiencing higher levels of comorbid psychopathology, than individuals who used cannabis, tobacco, and/or alcohol. Healey et al. (12) also focused on a treatment sample, finding that both cannabis users and their clinicians reported difficulty in establishing a therapeutic bond. A dose–response relationship was evident for the client perspective, such that heavier users reported feeling less connected, which the authors suggest may be related to effects of cannabis use such as paranoia or anxiety. The association between cannabis use and anxiety is explored by Temple et al. (13), who test the premise that the contradictory findings in the literature for this association may be due to individuals misattributing stress responses to anxiety symptomology. The finding that stated use to self-medicate for anxiety is more strongly associated with level of stress rather than anxiety symptoms provides some support for this hypothesis.

The therapeutic potential of cannabis is one of the factors driving the push for legalization of cannabis use. Yet, as discussed by Crippa et al. (14), with the majority of past research focus being on cannabis as a whole or THC, we have limited knowledge of the mechanisms of action of the many other cannabinoids, which is impeding our understanding of their medical applications. One of the key areas of current research into the therapeutic effects of cannabis focuses on the ability of CBD to modulate the adverse psychological effects of THC; this body of evidence is reviewed here by Niesink and van Laar (15). Oliere et al. (16) similarly focus on the therapeutic potential of cannabinoids, comprehensively reviewing what is known about the role of the endocannabinoid system in addiction and demonstrating the possibility of using cannabinoids to treat stimulant dependence. The focus on individual cannabinoids is also relevant to the issue of doping in sports, as is discussed by Bergamaschi and Crippa (17), who point out that focusing on THC metabolites for drug testing ignores the performance enhancing potential of other cannabinoids, such as CBD and CBN.

The final article in this research topic, by Burns et al. (18), urges researchers to reflect on the different indicators of cannabis use, demonstrating how the data we collect and inferences drawn will differ if we focus on the prevalence of cannabis use, for example, rather than the quantity of cannabis used or the frequency of use.

This article, along with the others collected here, encourage cannabis researchers to reflect on the ways in which we frame our research questions, design our studies, and explain our findings, so as to improve the clarity of the evidence. While we may not be able to clear the politicized smokescreen currently shrouding the evidence, ultimately, it is our responsibility to ensure that there is a comprehensive body of scientific knowledge available for the development of evidence-based cannabis policies and legislation when the fresh air does eventually blow through.

## Conflict of Interest Statement

The author declares that the research was conducted in the absence of any commercial or financial relationships that could be construed as a potential conflict of interest.
